# Supplementary material for: Actin cytoskeleton and complex cell architecture in an Asgard archaeon
Source: Nature. 2022 Dec 21;613(7943):332–9. doi: 10.1038/s41586-022-05550-y (PMC9834061; doi:10.1038/s41586-022-05550-y)
Supplement: Supplementary file 1 — Uncropped western blot shown in Fig. 5f. [file 41586_2022_5550_MOESM1_ESM.pdf]

nature portfolio

https://doi.org/10.1038/s41586-022-05550-y

Supplementary information

Actin cytoskeleton and complex cell architecture in an Asgard archaeon

In the format provided by the authors and unedited

Source Data Fig. 5f

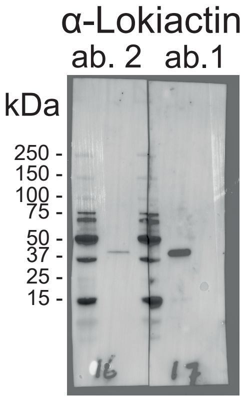

Supplementary Figure 1. Uncropped Western blot shown in Fig. 5f.
