# Supplementary material for: Actin cytoskeleton and complex cell architecture in an Asgard archaeon
Source: Nature. 2022 Dec 21;613(7943):332–9. doi: 10.1038/s41586-022-05550-y (PMC9834061; doi:10.1038/s41586-022-05550-y)
Supplement: Supplementary file 2 — Reporting Summary [file 41586_2022_5550_MOESM2_ESM.pdf]

nature portfolio

Corresponding author(s): Christa Schleper
Martin Pilhofer

Last updated by author(s): Oct 27, 2022

Reporting Summary

Nature Portfolio wishes to improve the reproducibility of the work that we publish. This form provides structure for consistency and transparency in reporting. For further information on Nature Portfolio policies, see our Editorial Policies and the Editorial Policy Checklist.

Statistics

For all statistical analyses, confirm that the following items are present in the figure legend, table legend, main text, or Methods section.

n/a	Confirmed	
☐	☒	The exact sample size (*n*) for each experimental group/condition, given as a discrete number and unit of measurement
☐	☒	A statement on whether measurements were taken from distinct samples or whether the same sample was measured repeatedly
☒	☐	The statistical test(s) used AND whether they are one- or two-sided<br>*Only common tests should be described solely by name; describe more complex techniques in the Methods section.*
☒	☐	A description of all covariates tested
☒	☐	A description of any assumptions or corrections, such as tests of normality and adjustment for multiple comparisons
☐	☒	A full description of the statistical parameters including central tendency (e.g. means) or other basic estimates (e.g. regression coefficient) AND variation (e.g. standard deviation) or associated estimates of uncertainty (e.g. confidence intervals)
☒	☐	For null hypothesis testing, the test statistic (e.g. *F*, *t*, *r*) with confidence intervals, effect sizes, degrees of freedom and *P* value noted<br>*Give P values as exact values whenever suitable.*
☒	☐	For Bayesian analysis, information on the choice of priors and Markov chain Monte Carlo settings
☒	☐	For hierarchical and complex designs, identification of the appropriate level for tests and full reporting of outcomes
☒	☐	Estimates of effect sizes (e.g. Cohen's *d*, Pearson's *r*), indicating how they were calculated

Our web collection on statistics for biologists contains articles on many of the points above.

Software and code

Policy information about availability of computer code

Data collection	CFX Maestro version 2.3 software for qPCR data collection; GRYPHAX version 1.1.8.153 for FISH imaging; SerialEM 4.0 for Cryo-ET data collection, Zeiss ZenBlue 3.5 for Airyscan data collection; Leica LASX for STED data collection
Data analysis	Fiji/ImageJ (v2.3.0/1.53q) for light microscopy analysis; IMOD (v4.11) for tomogram reconstruction and visualization; Dragonfly (v2022) for tomogram segmentation; RELION-4.0 for ribosome sub-tomogram averaging and actin reconstruction; ChimeraX (v1.3) for visualization; Dynamo for particle picking; R2DT for rRNA secondary structure prediction; ZenBlue 3.5 for Airyscan image processing; Huygens Professional (v22.04) for STED deconvolution; QIIME2 for amplicon analysis; Trimmomatic v.0.36 was used for trimming; Short reads were assembled with SPAdes v3.15.2; Binsanity, MaxBin2, MetaBAT, CONCOCT were used for genome binning; DAS tool for bin dereplication; Bonito 0.3.6 for nanopore reads basecalling; Porechop v0.2.4 and NanoFilt v2.8.0 for adapter removal and quality control of long reads; Flye v2.8.3-b1695 for assembly of long reads; Pilon for genome polishing; MAFFT v7.427 for sequence alignment; trimAl for alignment trimming; IQ-TREE 2.0 for phylogenetic tree reconstruction; BMGE for sequence alignment.

For manuscripts utilizing custom algorithms or software that are central to the research but not yet described in published literature, software must be made available to editors and reviewers. We strongly encourage code deposition in a community repository (e.g. GitHub). See the Nature Portfolio guidelines for submitting code & software for further information.

Data

Policy information about availability of data

All manuscripts must include a data availability statement. This statement should provide the following information, where applicable:

- Accession codes, unique identifiers, or web links for publicly available datasets
- A description of any restrictions on data availability
- For clinical datasets or third party data, please ensure that the statement adheres to our policy

The accession number of the genome is CP104013 (GenBank), under BioProject ID: PRJNA847409, BioSample accession: SAMN28933922.
Sub-tomogram averages (accession codes: EMD-15987 - EMD15988), example tomograms (accession codes: EMD-15989 - EMD-15993) and corresponding tilt-series (accession code: EMPIAR-11269) have been uploaded to the Electron Microscopy Databank or the Electron Microscopy Public Image Archive.
Other datasets used in this study from the Electron Microscopy Databank: EMD-13448, EMD-11976
Other datasets used in this study from the Protein Data Bank (PDB): 6SKF, 3J8A, 5MW1

Human research participants

Policy information about studies involving human research participants and Sex and Gender in Research.

Reporting on sex and gender	N/A
Population characteristics	N/A
Recruitment	N/A
Ethics oversight	N/A

Note that full information on the approval of the study protocol must also be provided in the manuscript.

Field-specific reporting

Please select the one below that is the best fit for your research. If you are not sure, read the appropriate sections before making your selection.

☒ Life sciences ☐ Behavioural & social sciences ☐ Ecological, evolutionary & environmental sciences

For a reference copy of the document with all sections, see nature.com/documents/nr-reporting-summary-flat.pdf

Life sciences study design

All studies must disclose on these points even when the disclosure is negative.

Sample size	No sample size calculations were performed. Imaging experiments were performed on samples derived from at least 2 independent cultures (as stated in the figure legends). For sub-tomogram averaging, please see ED Fig. 6 and 9 and the methods section for the number of particles and particle selection.
Data exclusions	Cultures were selected based on detectable exponential growth for experiments. No data were excluded from the analysis. For exclusion of particles from sub-tomogram averaging, please see ED Fig. 6 and 9 and the methods section.
Replication	FISH, RT-qPCR, SEM, TEM, IF and cryoET and immunogold localization were all performed from two to five independent times (as stated in the figure legends), with all attempts at replication being succesful.
Randomization	Randomization is not relevant for the current study as it does not involve participant groups.
Blinding	Blinding is not relevant to the present study, as it is cultivation based and the researchers involved need to to verify samples and controls for each experiment.

Reporting for specific materials, systems and methods

We require information from authors about some types of materials, experimental systems and methods used in many studies. Here, indicate whether each material, system or method listed is relevant to your study. If you are not sure if a list item applies to your research, read the appropriate section before selecting a response.

Materials & experimental systems

n/a	Involved in the study
☐	☒ Antibodies
☒	☐ Eukaryotic cell lines
☒	☐ Palaeontology and archaeology
☐	☒ Animals and other organisms
☒	☐ Clinical data
☒	☐ Dual use research of concern

Methods

n/a	Involved in the study
☒	☐ ChIP-seq
☒	☐ Flow cytometry
☒	☐ MRI-based neuroimaging

Antibodies

Antibodies used

Custom antibodies were obtained using the services of Eurogentec (order number 1000997858). They were produced in rabbits (ab1: CTFYTDLRVDPSEHPV - lot number 2110517; ab2: CSKNGFAGEDQPRSVF - lot number 2110516) and used at dilutions of either 1:100/1:500 (for IF) or 1:1000 (for WB).
Secondary antibodies for immunofluorescence and Western blotting experiments were: donkey anti-rabbit AF647, Invitrogen A-31573 (1:500 diluted), goat anti-rabbit abberior STAR 580, abberior ST580-1002 (1:200 diluted) and goat anti-rabbit HRP, Invitrogen 31460 (1:5000 diluted).

Validation

The identity of the peptides was confirmed by LC-MS analysis. The antibodies were validated through ELISA assays using the services of Eurogentec and additionally tested on L. ossiferum cell lysate by Western blotting (see Fig. 5f).

Animals and other research organisms

Policy information about studies involving animals; ARRIVE guidelines recommended for reporting animal research, and Sex and Gender in Research

Laboratory animals

For laboratory animals, report species, strain and age OR state that the study did not involve laboratory animals.

Wild animals

Provide details on animals observed in or captured in the field; report species and age where possible. Describe how animals were caught and transported and what happened to captive animals after the study (if killed, explain why and describe method; if released, say where and when) OR state that the study did not involve wild animals.

Reporting on sex

Indicate if findings apply to only one sex; describe whether sex was considered in study design, methods used for assigning sex. Provide data disaggregated for sex where this information has been collected in the source data as appropriate; provide overall numbers in this Reporting Summary. Please state if this information has not been collected. Report sex-based analyses where performed, justify reasons for lack of sex-based analysis.

Field-collected samples

Collection of the sediment samples and the enrichment culture are described in the Methods section.

Ethics oversight

Identify the organization(s) that approved or provided guidance on the study protocol, OR state that no ethical approval or guidance was required and explain why not.

Note that full information on the approval of the study protocol must also be provided in the manuscript.
